# Experimental Evidence for the Incorporation of Two Metals at Equivalent Lattice Positions in Mixed‐Metal Metal–Organic Frameworks

**DOI:** 10.1002/chem.201905596

**Published:** 2020-03-11

**Authors:** Johannes Bitzer, Steffen Otterbach, Kavipriya Thangavel, Anastasia Kultaeva, Rochus Schmid, Andreas Pöppl, Wolfgang Kleist

**Affiliations:** ^1^ Faculty of Chemistry and Biochemistry Industrial Chemistry – Nanostructured Catalyst Materials Ruhr University Bochum Universitätsstraße 150 44801 Bochum Germany; ^2^ Institute for Chemical Technology and Polymer Chemistry Karlsruhe Institute of Technology Engesserstr. 18/ 20 76131 Karlsruhe Germany; ^3^ Felix Bloch Institute for Solid State Physics Leipzig University Linnéstr. 5 04103 Leipzig Germany; ^4^ Faculty of Chemistry and Biochemistry Chair of Inorganic Chemistry 2 – Computational Materials Chemistry Ruhr University Bochum Universitätsstraße 150 44801 Bochum Germany

**Keywords:** copper, EXAFS, iron, metal–organic frameworks, X-ray absorption spectroscopy

## Abstract

Metal–organic frameworks containing multiple metals distributed over crystallographically equivalent framework positions (mixed‐metal MOFs) represent an interesting class of materials, since the close vicinity of isolated metal centers often gives rise to synergistic effects. However, appropriate characterization techniques for detailed investigations of these mixed‐metal metal–organic framework materials, particularly addressing the distribution of metals within the lattice, are rarely available. The synthesis of mixed‐metal FeCuBTC materials in direct syntheses proved to be difficult and only a thorough characterization using various techniques, like powder X‐ray diffraction, X‐ray absorption spectroscopy and electron paramagnetic resonance spectroscopy, unambiguously evidenced the formation of a mixed‐metal FeCuBTC material with HKUST‐1 structure, which contained bimetallic Fe−Cu paddlewheels as well as monometallic Cu−Cu and Fe−Fe units under optimized synthesis conditions. The in‐depth characterization showed that other synthetic procedures led to impurities, which contained the majority of the applied iron and were impossible or difficult to identify using solely standard characterization techniques. Therefore, this study shows the necessity to characterize mixed‐metal MOFs extensively to unambiguously prove the incorporation of both metals at the desired positions. The controlled positioning of metal centers in mixed‐metal metal–organic framework materials and the thorough characterization thereof is particularly important to derive structure–property or structure–activity correlations.

## Graphical abstract

**Two is company**: The incorporation of Fe and Cu on crystallographically equivalent positions in a phase‐pure mixed‐metal HKUST‐1 material was proven using X‐ray absorption spectroscopy and electron paramagnetic resonance spectroscopy. In particular, this study showed that the presence of small amounts of other phases, which seemed neglectable based on standard characterization techniques, must not be ignored, since they might contain a major amount of one of the used metals.

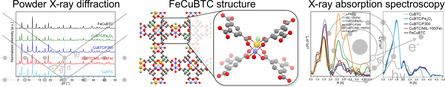

## Introduction

Metal–organic frameworks (MOFs), also known as porous coordination polymers, are a rather young class of materials. Within recent years, metal–organic frameworks gained growing interest, since they are promising materials for various applications like gas storage^1^ and separation,^2^ drug delivery^3^ and catalysis.^4^ They are built from inorganic secondary building units (SBUs), which are connected by rather rigid organic molecules (linkers) and form two‐ or three‐dimensional structures, often containing micro‐ and/or mesopores.

**Figure 1:**
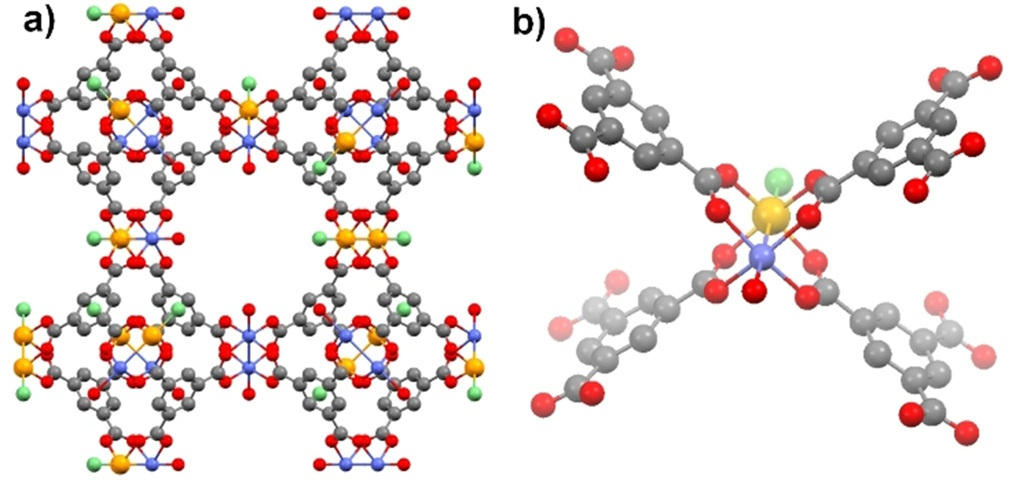
Schematic framework structure (a) and the characteristic paddlewheel unit (b) of a mixed‐metal FeCuBTC material with HKUST‐1 structure. Blue: Cu; orange: Fe; green: Cl; red: O; gray: C.

One of the most‐studied metal–organic framework materials is CuBTC (also known as HKUST‐1,^5^ MOF‐199^6^ or Cu_3_BTC_2_,^7^ see Figure 1 a), which contains dimeric copper units and benzene‐1,3,5‐tricarboxylate (BTC) as building blocks. The characteristic and well‐known SBU is a paddlewheel, which consists of a dimeric copper unit that is bridged by four carboxylate groups from four BTC molecules (see Figure 1 b). Usually, the two axial positions of the paddlewheels are occupied by water or other solvent molecules, but the removal of these molecules from the axial positions by heat and/or vacuum treatment leads to the formation of coordinatively unsaturated sites (CUS) at the copper atoms.^8^ These CUS can be reached by guest molecules entering the pores, which enables a direct metal‐guest interaction. Due to the possibility of direct metal‐guest interactions within the HKUST‐1 structure it is particularly interesting to change the type of metals used for the synthesis. Indeed, several other MBTC materials (M=Cr,^9^ Mn,^10^ Fe,^11^ Co,^10^ Ni,^12^ Zn,^13^ Mo,^14^ Ru^15^) with HKUST‐1 structure have been reported. Theoretical calculations proposed favorable oxygen adsorption properties for materials with HKUST‐1 structure containing early 3d transition metals (Sc or Ti), but such materials have not been synthesized so far.^16^

The combination of different types of metals in one framework can lead to outstanding new properties.^17^ Of special interest are mixed‐metal metal–organic framework materials, in which different metal types are distributed over crystallographically equivalent framework positions.^18^ By using this approach, the properties of a known and promising framework structure can be extended and altered without changing the framework topology. For the HKUST‐1 structure, some mixed‐metal structures with the metal combinations Cu/Ru,^19^ Cu/Zn,^20^ Cu/Ni,^21^ Cu/Pd,^22^ Cu/Ag,^23^ Cu/Mn,^24^ Cu/Fe^24^ and Cu/Co^24^ have already been reported. The first four have been synthesized via direct syntheses, while the latter ones used post‐synthetic metal exchange. The preparation of these mixed‐metal HKUST‐1 structures is expected to result in interesting new properties, since direct metal–metal interactions are possible within the bimetallic paddlewheel units. Theoretical calculations of Zhang et al. have shown that a mixed‐metal CuWBTC should have potential for the activation of CO_2_,^25^ although the synthesis and experimental verification of this claim has not been reported yet.

The development of synthesis routes for mixed‐metal MOFs, especially the generally favored direct synthesis, is often challenging, though. The influence of various parameters (e.g. crystallization rates, different preferred coordination geometries, competing structures or solubility issues) might lead to the formation of phase mixtures of different structures or phase mixtures of monometallic HKUST‐1 structures. Moreover, even completely new structures containing two types of metals might be obtained as undesired side products. Furthermore, a metal–organic framework structure with metal nanoparticles within the pores, as reported by Zhang et al.,^22^ is generally possible as well. Hence, a careful characterization of the synthesized materials is of high importance. For only a few of the reported mixed‐metal HKUST‐1 structures, the presence of bimetallic paddlewheel structures has been truly verified and the formation of other undesired phases has been unambiguously excluded.

Within this article, the development of a direct synthesis route for mixed‐metal FeCuBTC is reported. Furthermore, we present a detailed characterization with special attention on competing structures. While such impurities have been detected for the majority of the synthesized materials, the characterization data for a phase‐pure FeCuBTC material proved that iron was only incorporated within the paddlewheel units. Moreover, bimetallic paddlewheels containing copper and iron were found. The experimental proof for the incorporation of both metals in defined positions is important for all mixed‐metal metal–organic framework materials, but the technical realization is rather difficult, though.

## Results and Discussion

As a starting point for the synthesis of mixed‐metal FeCuBTC materials, various synthetic procedures were chosen that had been reported in the literature for CuBTC. These included solvothermal syntheses, syntheses at ambient pressure and microwave assisted syntheses. Furthermore, post‐synthetic metal exchange as reported by Sava Gallis et al.^24^ has been tested to synthesize an iron‐ and copper‐containing mixed‐metal HKUST‐1 material as reference sample, but no phase‐pure material could be obtained even after slight variations of the synthetic procedure.

All syntheses were performed with an Fe:Cu ratio of 30:70. In the majority of the obtained products, mixtures of different phases have been observed. The undesired side products could be clearly identified as α‐Fe_2_O_3_, MIL‐100(Fe) and Basolite F300 based on the powder X‐ray diffraction patterns. Particularly, syntheses that contained an excess of total metal amount with respect to 1,3,5‐benzenetricarboxylic acid showed significant amounts of iron‐rich phases in the diffraction patterns. However, several samples have been obtained that showed no or only minor reflections of undesired side products and these were initially assumed to be neglectable. In the following, three of these samples will be presented exemplary, which are denoted as CuBTC/Fe_2_O_3_, CuBTC/MIL‐100(Fe) and CuBTC/F300. CuBTC and MIL‐100(Fe) have been synthesized as phase‐pure materials and used as references together with commercially available α‐Fe_2_O_3_. Unfortunately, the initially intended synthesis of a FeBTC reference material with HKUST‐1 structure, as reported by Xie et al.,^11^ could not be reproduced. One of the synthesized samples, in the following denoted as FeCuBTC, did not show any impurities, even after a thorough characterization using various techniques (vide infra). Thus, we claim that this material is truly a mixed‐metal HKUST‐1 sample, which contains iron and copper distributed over equivalent metal framework positions, as will be shown in the following.

**Figure 2:**
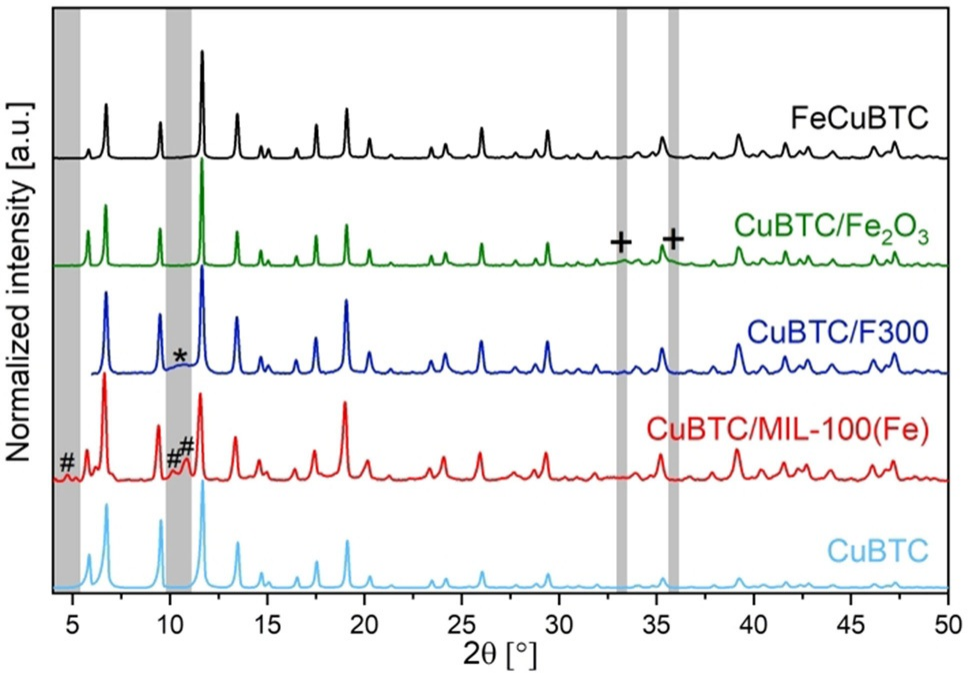
Powder X‐ray diffraction patterns of the synthesized Fe‐Cu‐BTC containing materials and CuBTC as reference. All diffraction patterns were normalized to the largest reflection of each material. Reflections, which do not belong to the HKUST‐1 structure, are indicated by + (α‐Fe2O3), * (F300) or # (MIL‐100(Fe)). For detailed views see Figure S**1**.

The powder X‐ray diffraction (PXRD) patterns (Figure 2) showed a mixture of phases for CuBTC/MIL‐100(Fe). Although the respective reflections of the MIL‐100(Fe) phase were weak compared to the reflections of the HKUST‐1 structure, they could be clearly identified. In the case of CuBTC/Fe_2_O_3_, only the high quality of the data up to 2*θ*=50 ° (cf. Figure S1) and a careful comparison with other characterization techniques that will be presented in this article, allowed the unambiguous identification of the α‐Fe_2_O_3_ phase. The corresponding weak reflections of α‐Fe_2_O_3_ could be identified at 2*θ*=33.4 ° and 35.9 ° and would have been easily missed if the noise level was more pronounced, a smaller 2 *θ* range was chosen for the measurement or the data were not analyzed thoroughly.

The identification of the F300 phase in CuBTC/F300 was also rather difficult and would have been easily missed on a first glance. This was due to the rather amorphous character of the F300 material, which did not show any sharp reflections in the PXRD pattern. Initially, the broad reflection centered at 2*θ*=10.7 ° was first interpreted as a part of the background signal originating from the sample holder and only further detailed characterization proved the presence of F300. Based on the powder X‐ray diffraction data, FeCuBTC showed no other reflections beside the HKUST‐1 structure, particularly none of the above‐mentioned ones. This indicated the formation of a phase‐pure material with solely HKUST‐1 structure. The Pawley fit of FeCuBTC (see Figure S2 and Table S1) showed that the incorporation of iron into the HKUST‐1 structure resulted in a slight expansion of the unit cell compared to CuBTC (26.323 Å vs. 26.313 Å).

Nitrogen physisorption measurements showed that all materials featured type I isotherms, which are typical for microporous solids. The specific surface areas of all mixed‐metal materials were in the range from 670 m^2^ g^−1^ (CuBTC/Fe_2_O_3_) to 1070 m^2^ g^−1^ (CuBTC/MIL‐100(Fe)) and, thus, significantly lower than the value for CuBTC (see Figure S3 and Table S3).

The metal ratios were determined using ICP‐OES analysis of digested samples (see Table S2). Both FeCuBTC and CuBTC/MIL‐100(Fe) showed the expected Fe:Cu ratio of 30:70, which had been applied during the synthesis. Although CuBTC/F300 and CuBTC/Fe_2_O_3_ were synthesized using the same initial ratio, the obtained product showed a significantly higher iron content (Fe:Cu≈40:60). This might be related to the fact that an excess of the metal precursors was used with respect to 1,3,5‐benzetricarboxylic acid. A time‐dependent study on the course of the reaction for a CuBTC/F300 related material showed that an iron‐rich F300 phase was formed in the beginning and only after several minutes an HKUST‐1 structure was observed, which explained the found metal ratios (see Figure S4 and Table S4).

**Figure 3:**
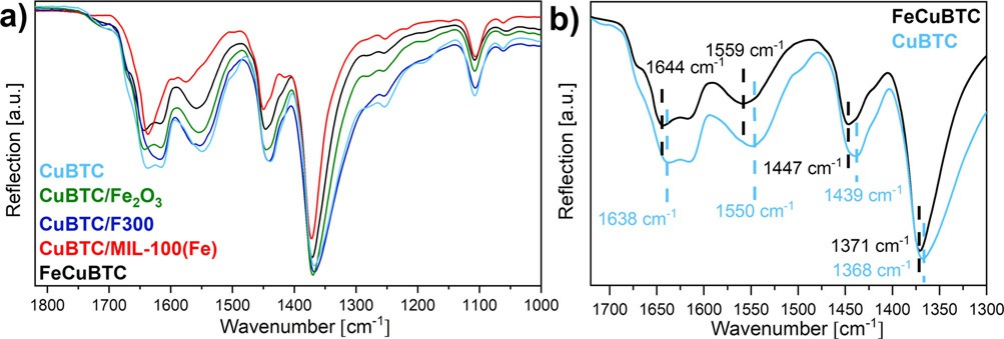
ATR‐IR spectra of the mixed‐metal materials compared to CuBTC. Carboxylate vibrations of all samples (a) and a comparison of FeCuBTC and CuBTC showing the shifts of these carboxylate bands (b).

The recorded ATR‐IR spectra proved the absence of residual linker molecules within the pores (carboxylate vibrations expected at 1700 cm^−1^) for all obtained materials (see Figure 3 a). Furthermore, the band pattern of all samples showed several similarities within this region and the main differences were variations in band intensities or small shifts of some bands. In particular, the strong similarity of FeCuBTC and CuBTC indicated similar coordination modes of the carboxylate groups within both materials. In addition, the similar spectrum of CuBTC/Fe_2_O_3_ might be reasonable, since the formed α‐Fe_2_O_3_ is not expected to have any significant bands in the measured range of the ATR‐IR spectra. Therefore, only the HKUST‐1 structure of CuBTC/Fe_2_O_3_ contributed to the observed IR spectrum. The presence of small shifts of FeCuBTC (e.g. from 1638 cm^−1^ to 1643 cm^−1^ and from 1550 cm^−1^ to 1559 cm^−1^, see Figure 3 b) compared to CuBTC indicated that the vibration frequency of the carboxylate vibration has changed compared to monometallic CuBTC. Possible reasons for this might be a different coordination mode of the carboxylate groups or the presence of different metals, to which the carboxylate groups are coordinated. The latter possibility would be reasonable if iron was present in the paddlewheel units instead of copper. The change of electronegativity from copper to iron would also influence the vibration frequency of the coordinated carboxylate groups. Moreover, a closer view on Figure 3 showed that small shifts were visible for CuBTC/Fe_2_O_3_ as well, which might indicate that iron has been partially used for the formation of α‐Fe_2_O_3_ and partially for the incorporation in the paddlewheels of the HKUST‐1 structure.

Density functional theory (DFT) calculations of non‐periodic model systems have been performed to evaluate whether the observed shifts of the IR bands are in a realistic range for bimetallic paddlewheel units. These calculations were performed for isolated paddlewheel units with four benzoate ligands. Three cases have been considered: (i) a Cu−Cu paddlewheel with two axial water molecules coordinated to the copper centers, (ii) a Cu−Fe paddlewheel with one axial water molecule coordinated to the copper center and one axial chloride ion coordinated to the iron center and (iii) a Fe−Fe paddlewheel with two chloride ions coordinated to the iron centers.

All structures were fully optimized using a hybrid functional DFT method (including dispersion corrections) until a true energy minimum was located (see Figure S5). Also, in the case of Fe‐containing paddlewheels, only slightly twisted structures were found, allowing the formation of the periodic MOF structure. Subsequently, a normal mode analysis was performed to calculate the IR vibrational frequencies and intensities (see Figure S6). The results showed that the positions of carboxylate vibrations were shifted upon the exchange of the metal centers in the same order of magnitude as observed experimentally and that the band positions of vibrations of the bimetallic Fe−Cu paddlewheel were in between those for the monometallic systems. However, the calculated direction of the shifts matched only partially with the experimentally observed ones. A possible reason for these differences could be an insufficient accuracy of the theoretical level for these small changes. In addition, the approximation of isolated paddlewheel units instead of a complete MOF structure might influence the results. Note that the considered modes were linker vibrations, which are only indirectly influenced by the type of the metal centers and, thus, the influences of the metal substitution are difficult to capture. M−O stretch vibrations would, therefore, be a better probe but are experimentally not accessible. Nonetheless, the performed theoretical calculations indicated that the metal substitution within the paddlewheel had an influence on the vibration frequency of the carboxylate groups, which, in combination with experimentally data, suggested that bimetallic paddlewheels were formed for the FeCuBTC material.

**Figure 4:**
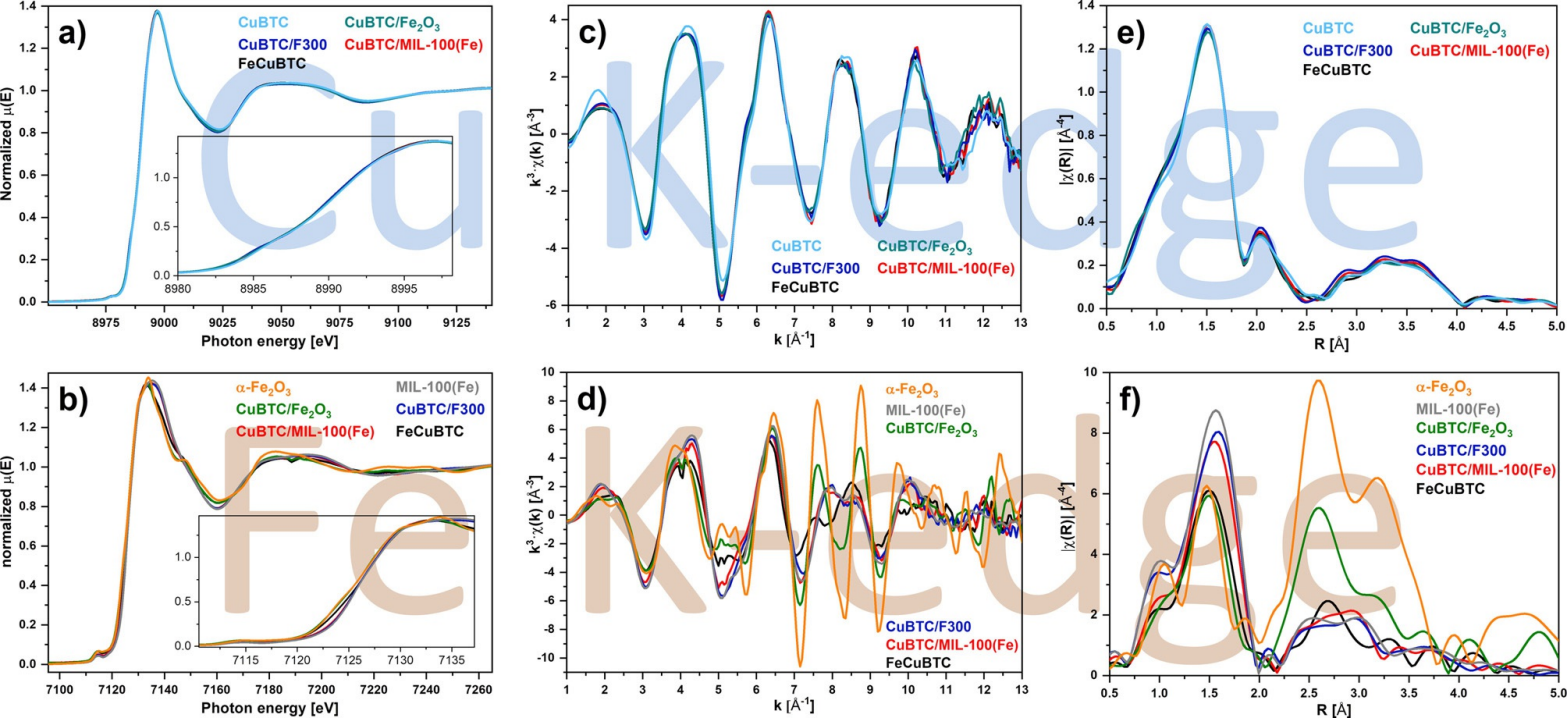
XANES spectra (a+b), EXAFS spectra (c+d) and Fourier‐transformed EXAFS spectra (e+f) recorded at the Cu K‐edge (a,c,e) and at the Fe K‐edge (b,d,f). No phase shift correction was applied.

In order to get a closer insight into the chemical environment on the atomic scale, X‐ray absorption spectroscopy measurements have been performed using synchrotron radiation at PETRA III, DESY (Hamburg). Only small differences were visible in the XANES region at the Cu K‐edge (see Figure 4 a) and the position and shape of the edges suggested that all samples contained copper in the +II oxidation state, which was in accordance with other literature reports.^8^, ^26^ The EXAFS and Fourier‐transformed EXAFS spectra (see Figure 4 c, e) showed small differences between the monometallic CuBTC and the mixed‐metal samples. All mixed‐metal samples looked alike, though. These observations might indicate the incorporation of minor amounts of iron next to copper within the paddlewheel units for all mixed‐metal samples. In the R‐space, the largest differences were visible in the region around *R*=2.4 Å, which would be a reasonable distance for a Cu−Fe dimer in a bimetallic paddlewheel unit if a phase shift correction is used.

The XANES spectra recorded at the Fe K‐edge showed clear differences (see Figure 4 b). The spectra of MIL‐100(Fe), CuBTC/MIL‐100(Fe) and CuBTC/F300 were nearly identical, but significantly different from those of CuBTC/Fe_2_O_3_ and the α‐Fe_2_O_3_ reference, which were also similar. On the other hand, the XANES spectrum of FeCuBTC looked different from all other samples. The oxidation state of iron was determined to be +III for all samples, though.^27^ A closer look at the EXAFS spectra (see Figure 4 d) led to similar conclusions. All samples containing a trimeric SBU (e.g. CuBTC/MIL‐100(Fe) and CuBTC/F300) looked identical and also the samples containing α‐Fe_2_O_3_ (e.g. α‐Fe_2_O_3_ and CuBTC/Fe_2_O_3_) were similar. For the latter ones, significant differences in the amplitude (*k*=7–10 Å^−1^) were observed. FeCuBTC was different from the other samples concerning the phase behavior as well as the amplitude, which supported the claim of a phase‐pure mixed‐metal FeCuBTC material, in which neither α‐Fe_2_O_3_ nor MIL‐100(Fe) or F300 was present.

MIL‐100(Fe), CuBTC/MIL‐100(Fe) and CuBTC/F300 differed from each other mainly in the intensity of the first shell in the Fourier‐transformed EXAFS spectra (see Figure 4 f). MIL‐100(Fe) showed the highest intensity, while CuBTC/MIL‐100(Fe) and CuBTC/F300 were less intensive. This observation was in accordance with the results at the Cu K‐edge, which indicated that minor amounts of iron might be incorporated in the HKUST‐1 structure for the latter two materials. In the MIL‐100(Fe) structure, iron is coordinated by six oxygen atoms, whereas it would be only coordinated by five oxygen atoms in a paddlewheel unit. If iron was present in both types of SBUs, the total intensity of the first shell would be lower compared to the pure MIL‐100(Fe). However, the overall similarity to MIL‐100(Fe) suggested that the majority of iron was present in a trimeric SBU similar to MIL‐100(Fe) and only minor amounts have been incorporated into the HKUST‐1 structure.

A closer look on α‐Fe_2_O_3_ and CuBTC/Fe_2_O_3_ showed that they differed in intensity of the higher shells (*R*=3–4 Å). The reduced intensity for CuBTC/Fe_2_O_3_ compared to pure α‐Fe_2_O_3_ might have two reasons; either the formed iron oxide particles were very small or a phase mixture, with iron being present as iron oxide and in a MOF structure at the same time, was formed. The first possibility could be ruled out, since reflections of the iron oxide phase were observed in the PXRD patterns (see Figure 2), which would not be the case for very small particles showing a reduced intensity of the corresponding shells. Therefore, in agreement with the above‐mentioned data, the presence of iron in α‐Fe_2_O_3_ and a MOF structure seemed plausible. Due to the dominant presence of light backscatters and, compared to iron oxide, a small number of total backscatterers around the iron center, a small intensity for shells for *R*>2 Å is expected for SBUs of the treated MOF structures. On the other hand, a comparable large number of iron backscatterers in the iron oxide phase results in high intensities in the range *R*=3–4.5 Å (see Figure 4 f). The combination of both situations, which will be effectively measured in XAS for phase mixtures, resulted in a reduced intensity for the higher shells as observed for CuBTC/Fe_2_O_3_. The identification or extraction of the MOF structure based on these EXAFS data would be a challenging task and the data quality would not be sufficient for these purposes. Based on these results, CuBTC/Fe_2_O_3_ might be comparable to the Pd@[Cu_3−*x*_Pd_x_(BTC)_2_]_*n*_ materials reported by Zhang et al.^22^ in the form of Fe_2_O_3_@[Cu_3−*x*_Fe_x_(BTC)_2_]_*n*_.

FeCuBTC differed significantly from all the mentioned samples in the R‐space. The intensity of the higher shells seemed plausible for a SBU of a metal–organic framework structure. The lower intensity of the first shell compared to MIL‐100(Fe) was also plausible for an HKUST‐1 structure, since only five nearest neighbors are expected for a paddlewheel structure instead of six for MIL‐100(Fe).

The Fourier‐transformed spectra were further analyzed by multi‐shell structure fitting. Since the SBU of these MOF materials contain several shells with light backscatterers, which are closer to the absorber atom than the next metal shell, several restraints had to be applied to the fitting models (in particular the carbon and oxygen shells) to assure a reasonable number of free variables for the applied number of fitted shells.

The analysis of the Fourier‐transformed EXAFS spectra showed that the obtained spectrum for CuBTC corresponded closely to the reported single crystal structure (see Figure S7 and Table 1). The first shell corresponded to five oxygens in a distance of 1.95 Å. Four of them originated from coordinated carboxylate groups, the fifth from coordinated water at the axial position of the paddlewheel. The second copper atom of the copper dimer in the paddlewheel unit was found at 2.65 Å and four carbon neighbors at 2.86 Å were needed to obtain a reasonable fit quality. Higher shells could not be resolved with sufficient accuracy.

**Table 1:** Best fit values for CuBTC and FeCuBTC.

Sample	Abs−Bs^[a]^	N(Bs)^[b]^	R(Abs−Bs)^[c]^ [Å]	σ^2[d]^ [Å]
**HKUST‐1**	Cu−O	5	1.95±0.02	0.0061±0.0005
Cu K‐edge	Cu−Cu	1	2.67±0.06	0.0160±0.0088
	Cu−C	4	2.88±0.06	0.0160±0.0088
general fitting parameters:	*S*_0_^2[e]^=0.8892; Δ*E*_0_^[f]^=5.71±1.22 eV; χ^2^_red_^[g]^=915; *R* ^[h]^=0.019; *N*(path)^[i]^=3; *N*(par)^[j]^=5; *k*‐range: 3‐13; *R*‐range: 1.0–3.0.			
**FeCuBTC**	Cu−O	5	1.96±0.02	0.0061±0.0004
Cu K‐edge	Cu−Cu	0.57±0.10*	2.62±0.05	0.0153±0.0068
	Cu−Fe	0.43±0.10*	2.75±0.09	0.0153±0.0068
	Cu−C	4	2.83±0.05	0.0153±0.0068
	Cu−O	4	3.08±0.04	0.0153±0.0068
general fitting parameters:	*S*_0_^2[e]^=0.8892; Δ*E*_0_^[f]^=6.88±1.25 eV; χ^2^_red_^[g]^=290; *R* ^[h]^=0.009; *N*(path)^[i]^=5; *N*(par)^[j]^=8; *k*‐range: 3‐13; *R*‐range: 1.0–3.0.			
**FeCuBTC**	Fe−O	4	1.95±0.02	0.0060±0.0010
Fe K‐edge	Fe−Cl	1	2.24±0.05	0.0085±0.0037
	Fe−Cu	0.58±0.31*	2.79±0.05	0.0060±0.0010
	Fe−C	4	3.02±0.03	0.0060±0.0010
	Fe−Fe	0.42±0.31 *	3.03±0.03	0.0060±0.0010
	Fe−O	4	3.28±0.04	0.0060±0.0010
general fitting parameters:	*S*_0_^2[e]^=0.7592; Δ*E*_0_^[f]^=−4.57±4.37 eV; χ^2^_red_^[g]^=192; *R* ^[h]^=0.035; *N*(path)^[i]^=6; *N*(par)^[j]^=9; *k*‐range: 3‐13; *R*‐range: 1.0–3.1.			

*Coordination number was used as fit parameter or obtained from a parameter optimized in the fit. [a] Abs=X‐ray absorbing atom, Bs=backscattering atom. [b] Number of backscattering atoms. [c] Distance between absorbing and backscattering atom. [d] Debye‐Waller factor. [e] Amplitude reduction factor. [f] Accounts for the shift of E_0_ between theory and experiment. [g] Reduced χ^2^ error (considers the number of independent points and number of varied parameters besides the error to the experiment). [h] Fit index. [i] Total number of fitted paths including single and multiple scattering paths. [j] Number of free parameters used for the fit.

**Figure 5:**
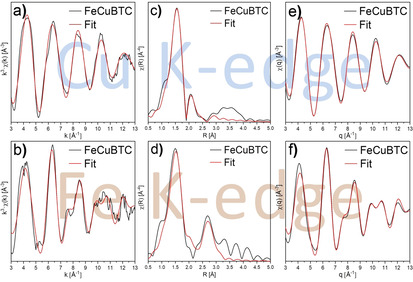
Best fit calculations and experimentally obtained EXAFS spectra (a+b), Fourier‐transformed EXAFS spectra (c+d) and Fourier back‐transformed spectra (e+f) of FeCuBTC at the Cu K‐edge (a,c,e) and the Fe K‐edge (b,d,f).

A similar fit was performed on the Fourier‐transformed EXAFS spectrum of FeCuBTC at the Cu K‐edge, but worse results have been obtained, especially at *R*=2.4 Å (see Figure S8 and Table S5). By adding an iron shell in addition to the already fitted copper shell, the results could be improved (see Figure 5 a, c, e and Table 1). The total number of iron and copper neighbors was fixed to one, but the Fe:Cu ratio was used as a free fitting parameter and resulted in 0.57±0.10 copper neighbors and 0.43±0.10 iron neighbors. These results indicated that both monometallic copper paddlewheels and bimetallic iron‐copper paddlewheels were present in FeCuBTC, with nearly twice as much bimetallic paddlewheel units. Based on the obtained coordination numbers at the Cu K‐edge, the calculated Fe:Cu ratio of 30:70 is in accordance with the ICP‐OES measurements (see Table S2).

For the MIL‐100(Fe) reference sample, the experimental data at the Fe K‐edge were in accordance with the fitted data based on the structure of MIL‐100(Fe) obtained from single crystal analysis (see Figure S9 and Table S6). Similar fits were obtained for the samples CuBTC/MIL‐100 and CuBTC/F300 (see Figure S10 and Table S7), supporting the above presented assumptions that iron was present almost exclusively in the trimeric SBU that is typical for the MIL‐100(Fe) and F300 structures. In the case of α‐Fe_2_O_3_, multi‐shell fitting still represents a difficult task, because the number of paths in the range *R*=3–4.5 Å, is too large for reliable results. The same situation was present for CuBTC/Fe_2_O_3_, since not only a large amount of parameters would be necessary for α‐Fe_2_O_3_, but also for the MOF phase. Therefore, only the qualitative analysis presented above was possible.

The multi‐shell structure fitting of FeCuBTC at the Fe K‐edge gave unexpected and interesting insights into the local structure around the iron centers. During the data treatment, an inherent problem with the intensity of the first shell occurred, which could only be solved if either the number of nearest oxygen neighbors was increased to more than six or the amplitude reduction factor was increased to more than 1.0. Both options are physically not meaningful. Thus, an element with a higher atomic number than oxygen needed to be present close to iron. Since the oxidation state of iron was determined to be +III and the chloride salt was used during synthesis, a chloride ion seemed plausible, particularly due to the negative charge necessary to assure charge neutrality of the paddlewheel unit. Indeed, the fitting procedure with chloride at the axial position of the paddlewheel and four oxygens from the carboxylate groups returned the best results (see Figure S11 and Table S8). Furthermore, a fit with the first two oxygen shells from α‐Fe_2_O_3_ did not result in a reasonable fit (see Figure S12 and Table S9), which excluded the presence of iron in α‐Fe_2_O_3_ nanoparticles.

Further shells (C, O, Fe) were added according to the reported structure of FeBTC.^11^ The resulting fit was acceptable, but still major deviations were visible in the R‐space (*R*=2.4 Å) and q‐space (*q*=10.7 Å^−1^, see Figure S13 and Table S10). In the R‐space, these deviations were in a similar region like at the Cu K‐edge. By adding a copper shell in this region, a good fit was obtained (see Figure 5 b, d, f and Table 1). As already mentioned for the Cu K‐edge, the total number of metal neighbors was fixed to one and the Fe:Cu ratio was used as free parameter. The fit resulted in 0.58±0.31 copper neighbors in a distance of 2.79 Å and 0.42±0.31 iron neighbors in a distance of 3.03 Å. These results indicated the presence of both bimetallic Fe−Cu and monometallic Fe−Fe paddlewheel units. However, the number of the metal neighbors did not fully match with the found metal ratios by ICP‐OES. The complexity of the local structure might be a possible explanation. Due to a mixture of monometallic and bimetallic paddlewheel units, which differ in their metal–metal bond distances, a distortion of the local structure is expected for the bimetallic paddlewheel units resulting in numerous backscattering paths with slightly different distances for oxygen and carbon atoms. Therefore, this situation is too complex to be solved with high accuracy by EXAFS structure fitting based on the present data. The overlap of the Fe−Fe shell with C and O shells provides additional challenges in the fitting procedures, which leads to large errors in coordination numbers.

The EXAFS analysis at both the Fe K‐ and the Cu K‐edge of FeCuBTC clearly showed that bimetallic paddlewheel units were present. Furthermore, it indicated the presence of monometallic paddlewheels consisting of only copper or only iron. At both edges, only the assumption of bimetallic paddlewheel units resulted in good fit results.

**Figure 6:**
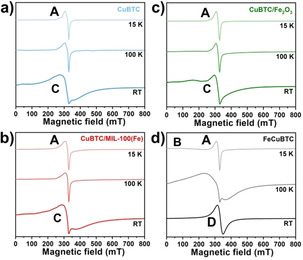
EPR spectra of CuBTC (**a**), CuBTC/MIL‐100(Fe) (**b**), CuBTC/Fe_2_O_3_ (**c**) and FeCuBTC (**d**) recorded at different temperatures.

Electron paramagnetic resonance (EPR) spectroscopy has been successfully used to investigate paddlewheel‐type structures in previous studies.20b, 28 Therefore, EPR spectra have been recorded for all materials to further corroborate the results (see Figure 6 and Table S11). All samples showed comparable EPR spectra at 15 K displaying the signal of monomeric Cu^2+^ species (**A**) with partly resolved Cu hyperfine (hf) splitting (parameters of **A**: *g*_zz_=2.340, *g*_xx/yy_=2.05, *A*_zz_=485 MHz). These features might be assigned to defective Cu−Cu paddlewheel units or extra‐framework cupric ions. Only for FeCuBTC, an additional broad baseline signal (**B**) was observed. The *g*_xx/yy_ spectral position at 328 mT of signal **A** was present in the spectra recorded at 100 K for all samples and at RT for CuBTC, CuBTC/MIL‐100(Fe), and CuBTC/Fe_2_O_3_. In CuBTC at RT, an additional broad signal (**C**) with a line width of about 80 mT and an isotropic *g*‐value of *g*=2.149 was observed, which could be assigned to the excited *S*=1 state of the antiferromagnetically coupled Cu^2+^ ions of the Cu−Cu paddlewheel units.28b Presumably, the signal observed for CuBTC/MIL‐100(Fe) at RT displaying a comparable line width might be likewise assigned to the Cu−Cu paddlewheel although the g‐value was somewhat lower. In the case of CuBTC/Fe_2_O_3_, the line width of this signal was considerably smaller and an assignment to the Cu−Cu paddlewheel was questionable although its g‐value was close to that of CuBTC.

FeCuBTC exhibited a distinct spectrum at RT. Here, an intense EPR signal (**D**) with a smaller *g*‐value (*g*=2.023) and linewidth (36 mT) was observed. The total EPR signal intensity at room temperature was higher in comparison to the three other samples (see Table S11). Neither signal **A** of the monomeric Cu^2+^ species nor signal **C** of Cu−Cu paddlewheel units were detected for FeCuBTC at RT. Moreover, the resolved fine structure signals of the *S*=1 state of the Cu−Cu paddlewheels,28b which are typically observed at 20 mT and 474 mT at 100 K, were not detected. Based on its g‐value, signal **D** cannot be assigned to either monomeric Cu^2+^ species or Cu^2+^ pairs in the paddlewheel unit. Therefore, we suggest that it was related to Cu^2+^−Fe^3+^ species in these samples. In addition, a comparison of the spectra of FeCuBTC recorded at RT and 100 K revealed that signal **D** did not disappear with decreasing temperature but exhibited a pronounced line broadening. Consequently, the broad baseline signal (**B**) recorded at 15 K for this sample might likewise be assigned to Cu^2+^−Fe^3+^ species. Note that high‐spin Fe^3+^ species in highly symmetric octahedral or tetrahedral environment would also provide an isotropic signal at *g*=2.00. However, the disappearance of signal features for FeCuBTC, which would indicate Cu−Cu paddlewheel units, suggested that signal **D** was not related to such Fe^3+^ species, but would instead indicate coupled Cu^2+^−Fe^3+^ moieties. Furthermore, the substantially larger overall EPR signal intensity of FeCuBTC in comparison with CuBTC, CuBTC/MIL‐100(Fe) and CuBTC/Fe_2_O_3_ supported this interpretation that a novel EPR active species was formed in FeCuBTC, which was not present in the other three materials.

Although the exact nature of these Cu^2+^−Fe^3+^ species could not be derived from these experiments, its g‐value and temperature dependence revealed some of its characteristics. The assumption of strongly exchanged coupled Cu^2+^−Fe^3+^ pairs and the coupling between the Cu^2+^ electron spin (*S*_Cu_=1/2) and the iron electron spin (*S*_Fe_=5/2) results in two spin states of the pair, *S*=2 and *S*=3. According to Buluggiu,^29^ the *S*=3 state provides a g‐value of *g*_pair_=2.00, which was in reasonable agreement with the measured *g*‐parameters of signal **D** if typical isotropic *g*‐values of 2.15 and 2.00 are assumed for Cu^2+^ and Fe^3+^. The isotropic nature of the signal D was indicative for strong magnetic exchange interactions among the Cu^2+^−Fe^3+^ species. The strong line broadening with decreasing temperature was astonishing and atypical for paramagnetic systems, but such effects are common for magnetic materials.^30^

## Conclusions

In summary, the presented results provide a detailed insight into the synthesis of a mixed‐metal HKUST‐1 structure containing copper and iron. A large variety of reaction parameters was screened and the obtained phases and phase mixtures were characterized thoroughly. It was found that a careful in‐depth characterization of the obtained mixed‐metal materials and a comparison to other iron‐ and copper‐containing materials was necessary to undoubtedly confirm the formation of a truly phase‐pure FeCuBTC material. In particular, the characterization using EXAFS analysis provided the necessary insight on the atomic scale to verify the presence of solely paddlewheel units as SBUs. The recorded EPR spectra further supported the hypothesis of bimetallic paddlewheels for FeCuBTC. Furthermore, this study showed that small amounts of impurities should not be assumed to be neglectable when working with mixed‐metal metal–organic framework materials, since the majority of one metal might be present exclusively within this impurity phase. Moreover, we demonstrated that a thorough characterization with high‐quality data is particularly important to unambiguously verify the formation of phase‐pure materials and the absence of any undesired phases in mixed‐metal MOF materials.

## Experimental Section

**Synthesis of FeCuBTC**: Cu(NO_3_)_2_⋅3 H_2_O (0.49 g, 2.04 mmol, 0.88 equiv.), FeCl_3_⋅6 H_2_O (0.24 g, 0.87 mmol, 0.37 equiv.), 1,3,5‐benzenetricarboxylic acid (H_3_BTC, 0.49 g, 2.33 mmol, 1.00 equiv.), 1,4‐diazabicyclo[2.2.2]octane (DABCO, 0.54 g, 4.80 mmol, 2.06 equiv.) and NaOH (0.09 g, 2.33 mmol, 1.00 equiv.) were weighed into a teflon vessel of a microwave sample tube. DMF (12.5 mL) and water (11.5 mL) were added and the vessel was sealed. The reaction mixture was heated to 140 °C within 5 min in a microwave oven and kept there for one hour. After cooling, the sample was filtered off, washed with DMF (3×20 mL) and water (1×20 mL), dried at room temperature overnight and for three days at 130 °C in air.

**Synthesis of CuBTC/MIL‐100(Fe)**: Cu(NO_3_)_2_⋅3 H_2_O (0.49 g, 2.04 mmol, 0.88 equiv.) and FeSO_4_⋅7 H_2_O (0.24 g, 0.87 mmol, 0.37 equiv.) were dissolved in water (10 mL). 1,3,5‐benzenetricarboxylic acid (H_3_BTC, 0.49 g, 2.33 mmol, 1.00 equiv.) was dissolved in a mixture of DMF (25 mL) and water (15 mL) at 100 °C. Both solutions were combined under stirring and kept at 100 °C under reflux cooling for two days. The sample was filtered off, washed with DMF (3×20 mL) and water (1×20 mL), dried at room temperature overnight and for three days at 130 °C in air.

**Synthesis of CuBTC/F300**: In a typical synthesis 1,3,5‐benzenetricarboxylic acid (H_3_BTC, 0.4896 g, 2.33 mmol, 1.00 equiv.) was dissolved in *N*,*N*‐dimethylformamide (DMF, 25 mL) at 100 °C. Iron(III) chloride hexahydrate (0.3925 g, 1.45 mmol, 0.62 equiv.) and copper(II) nitrate trihydrate (0.8185 g, 3.39 mmol, 1.45 equiv.; 4.84 mmol or 2.08 equiv. total metal amount) were dissolved in demineralized water (25 mL) at room temperature. Both solutions were combined and stirred at 100 °C for four hours. Afterwards the solid was filtered off using a glass filter and washed with 3×20 mL of DMF and 1×20 mL of demineralized water. The filtered product was dried in air at room temperature overnight and for another three days at 130 °C in an oven.

**Synthesis of CuBTC/Fe_2_O_3_**: Cu(NO_3_)_2_⋅3 H_2_O (0.49 g, 2.04 mmol, 0.88 equiv.), FeCl_3_⋅6 H_2_O (0.24 g, 0.87 mmol, 0.37 equiv.), 1,3,5‐benzenetricarboxylic acid (H_3_BTC; 0.49 g, 2.33 mmol, 1.00 equiv.), 1,4‐diazabicyclo[2.2.2]octane (DABCO, 0.54 g, 4.80 mmol, 2.06 equiv.) were weighed into a teflon vessel of a microwave sample tube. DMF (12.5 mL) and water (11.5 mL) were added and the vessel sealed. The reaction mixture was heated to 140 °C within 5 min in a microwave oven and kept there for two hours. After cooling, the sample was filtered off, washed with DMF (3×20 mL) and water (1×20 mL), dried at room temperature overnight and for three days at 130 °C in air.

**Powder X‐ray diffraction**: Powder X‐ray diffraction patterns were recorded using either a Bruker D8 Advance or a PANalytical Empyrean diffractometer both with Bragg–Brentano geometry. The samples were analyzed in the range of 2 *θ*=4–50 ° by using Cu_Kα_ radiation. A step width of 2 *θ*=0.0164 (Bruker) or 0.013° (PANalytical) was used. For visualization, a background subtraction with subsequent smoothing of the obtained diffraction patterns was performed. In order to compare measurements of the two different diffractometers, the obtained diffraction patterns were normalized with respect to the highest reflection of each pattern.

**X‐ray absorption spectroscopy**: XAS experiments were performed at PETRA III Extension beamline P65 (energy range: 4–44 keV) at DESY (Deutsches Elektronensynchrotron) in Hamburg (Germany). For the measurements at the Cu K‐ and/or Fe K‐edges, a Si(111) C‐type double crystal monochromator was used. The beam current was 100 mA with a ring energy of 6.08 GeV. All samples were prepared as pellets using cellulose as a binder. All spectra were recorded in continuous scan mode in transmission and fluorescence mode at ambient temperature and pressure in the range of −150 eV to 1000 eV around the edge in 180 sec. For the data analysis, transmission data were used. For calibration, the corresponding metal foils were measured as a reference simultaneously with the samples.

The data treatment was performed using the Demeter software package.^31^ In order to compensate for the oversampling of the continuous scan mode, the data points of the obtained spectra were reduced with the help of the “rebin” function of the Athena software (edge region: −50 to +50 eV; pre‐edge grid: 5 eV; XANES grid: 0.5 eV; EXAFS grid: 0.05 Å^−1^). For data evaluation, a Victoreen‐type polynomial was subtracted from the spectrum to remove the background using the Athena software. The first inflection point was taken as energy E_0_.

The EXAFS analysis was performed using the Artemis software. As starting models, cif‐files of CuBTC^5^ and FeBTC^11^ were used to generate the paths. Both, the k and R ranges were selected based on the data quality. Prior to the fitting procedure, the amplitude reduction factor S_0_^2^ was determined on a metal reference foil at the corresponding edge and used as fixed parameter for the samples, respectively. For the fitting procedure, several restraints had to be applied to obtain reasonable results. Therefore, most of the coordination numbers were fixed, Debye‐Waller factors were assumed to be the same for several neighbors and, in some cases, the variations of distances (ΔR) were coupled for shells that were close to each other.

**EPR measurements**: The EPR spectra were measured on a BRUKER EMXmicro X‐band spectrometer using an Oxford instruments ESR900 cryostat. As all spectra were found to be broadened, the modulation amplitude has been maintained as 10 G for the EPR measurements. The microwave power has been kept as either 2 mW or 0.2 mW depending on the signal quality. EPR measurements have been recorded at room temperature (RT), 100 K and 15 K.

**DFT calculations**: The frequency analysis for the three non‐periodic model systems (benzoate paddle‐wheels with Cu_2_, FeCu and Fe_2_ metal dimers, axial positions coordinated with H_2_O in case of Cu^2+^ and Cl^−^ in case of Fe^3+^; see Figure S5) were performed using the B3LYP+D3^32^ level of theory with cc‐PVDZ basis sets for C, H, O, and Cl and cc‐pVTZ for Cu and Fe.^33^ In all cases, the high‐spin ferromagnetic coupled state was used (Cu_2_: *S*=1, CuFe: *S*=3, Fe_2_: *S*=5) as indicated by the EPR results. In case of Cu_2_, the triplet state is energetically slightly above the open shell singlet but shows virtually the same normal modes.^34^ The optimization without any symmetry constraints and the analytic computation of the Hessian matrix have been performed with the TURBOMOLE (V7.3) suite of programs.^35^

## Conflict of interest

The authors declare no conflict of interest.

## Supporting information

As a service to our authors and readers, this journal provides supporting information supplied by the authors. Such materials are peer reviewed and may be re‐organized for online delivery, but are not copy‐edited or typeset. Technical support issues arising from supporting information (other than missing files) should be addressed to the authors.

Supplementary
