# Supplementary material for: Experimental Evidence for the Incorporation of Two Metals at Equivalent Lattice Positions in Mixed‐Metal Metal–Organic Frameworks
Source: Chemistry. 2020 Mar 11;26(25):5667–75. doi: 10.1002/chem.201905596 (PMC7317703; doi:10.1002/chem.201905596)
Supplement: Supplementary file 1 — Supplementary [file CHEM-26-5667-s001.pdf]

Chemistry–A European Journal

Supporting Information

Experimental Evidence for the Incorporation of Two Metals at Equivalent Lattice Positions in Mixed-Metal Metal–Organic Frameworks

Johannes Bitzer,[a] Steffen Otterbach,[b] Kavipriya Thangavel,[c] Anastasia Kultaeva,[c] Rochus Schmid,[d] Andreas Pöppl,[c] and Wolfgang Kleist*[a]

1. Experimental procedures

All chemicals have been bought and used as received without further purification. For microwave assisted syntheses, a Multiwave 3000 microwave reaction system from Anton Paar has been used. It was equipped with a sample rotor for up to eight samples and the temperature was controlled by automatically adjusting the microwave power based on a thermo couple placed inside one of the reaction vessels. A constant temperature for all vessels throughout the whole reaction was checked by an IR sensor, which measured the temperature on the outside wall of the Teflon inset of the reaction vessels.

Synthesis of CuBTC: The CuBTC sample was synthesized using a modified procedure reported by Marx *et al.*[1] In a typical synthesis, 1,3,5-benzenetricarboxylic acid (H_3BTC, 0.4896 g, 2.33 mmol, 1.00 eq.) was dissolved in N,N-dimethylformamide (DMF, 25 mL) at 100 °C. Copper(II) nitrate trihydrate (1.0824 g, 4.84 mmol, 2.08 eq.) was dissolved in demineralized water (25 mL) at room temperature. Both solutions were combined and stirred at 100 °C for 24 hours. Afterwards, the resulting solid was filtered off using a glass filter and washed with 3 x 20 mL DMF and 1 x 20 mL demineralized water. The filtered product was dried in air at room temperature over night and for another three days at 130 °C in an oven.

Synthesis of MIL-100(Fe): H_3BTC (0.1681 g, 0.80 mmol, 1.00 eq.), 1,4-diazabicyclo[2.2.2]octane (DABCO, 0.1795 g, 1.60 mmol, 2.00 eq.), iron(III) chloride hexahydrate (0.2162 g, 0.80 mmol, 1.00 eq.) and DMF (12 mL) were mixed in a teflon vessel, which was then placed in a sealed autoclave (Parr Instrument, 23 mL). The autoclave was heated to 150 °C for three days and then cooled down to room temperature within two hours. The solid product was filtered off with a glass filter and washed with 3 x 20 mL DMF (100 °C). The obtained product was dried in air at room temperature and for three days at 130 °C in an oven.

Structure refinements: The structure refinement was performed using TOPAS 4.2. The complete measured range from $2\theta = 4$ to 50 ° was used for the refinement. For all samples, the Pawley method was used to determine the unit cell parameters starting from literature values of CuBTC and FeBTC.

Infrared spectroscopy: A Vertex70 FT-IR spectrometer from Bruker Optics was used for recording ATR-IR spectra. The measurement cell was a Golden Gate Single Reflection Attenuated Total Reflection from Specac. All spectra were collected between 4500 cm^{-1} and 600 cm^{-1} using a resolution of 4 cm^{-1} and a MCT detector operating at liquid nitrogen temperature. For each measurement, 400 spectra were accumulated.

Nitrogen physisorption: The samples were activated for 20 h at 130 °C prior to the nitrogen physisorption measurements. The measurements were performed using a Belsorp mini II from BEL Japan. The specific surface areas were determined using the BET method (Brunauer, Emmet, Teller) and the BEL Master software.[2]

Elemental analysis: Metal ratios were determined using ICP-OES (inductively coupled plasma optical emission spectroscopy). An iCAP 6500 Duo from Thermo Scientific, Dreieich, Germany with standard equipment was used for the measurements. A six point standard was used for the calibration curve. Prior to measurement, the samples were dissolved in diluted nitric acid. The data analysis was performed with the device's own software 'iTEVA9.8'.

DFT calculation and frequency analysis:
DFT calculations have been performed using the TURBOMOLE program package as explained in the end of the Experimental Section in the main paper. The resolution of identity (RIDFT) approximation with the default additional bases for the electron density were used. A finer *m5* integration grid for the XC-interaction was used. For the D3 dispersion correction a Becke-Johnson damping was used.

2. Supplementary PXRD patterns

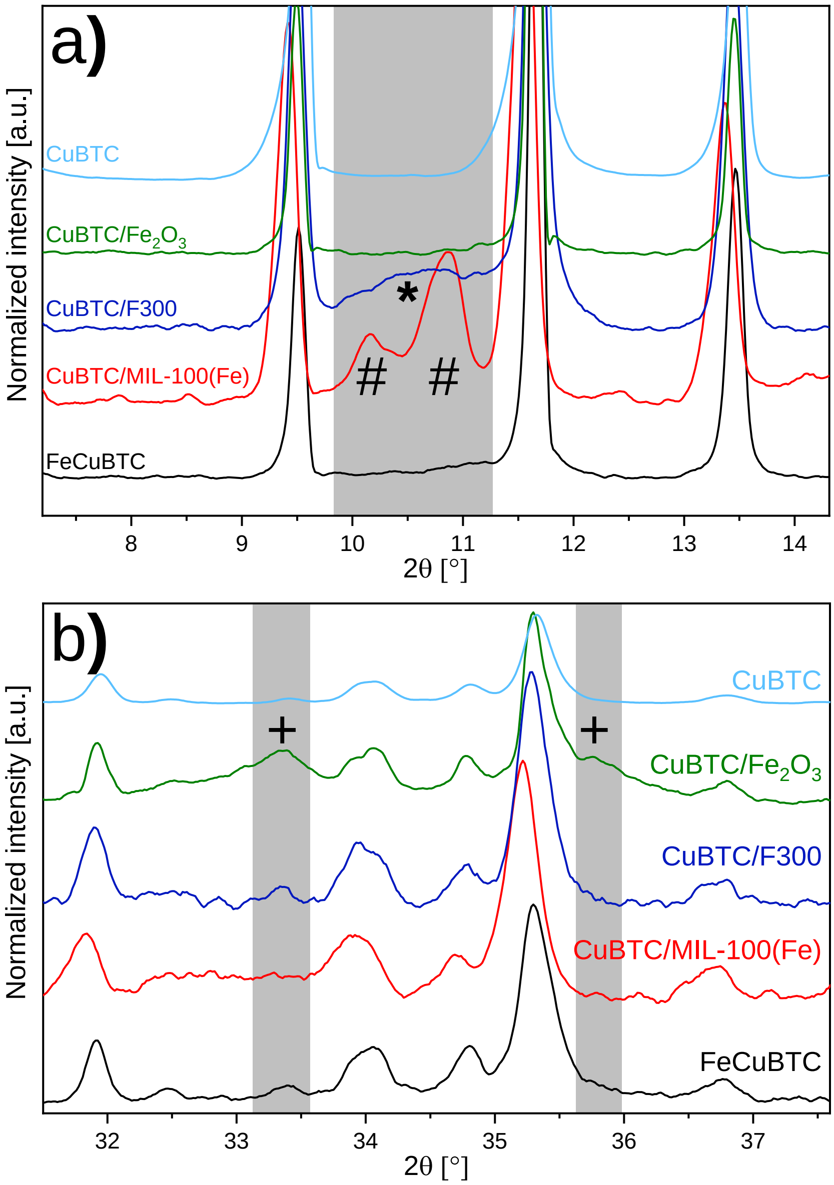

Figure S1. Detailed views of the recorded powder X-ray diffraction patterns presented in **Figure 2**.

3. Structure refinement results

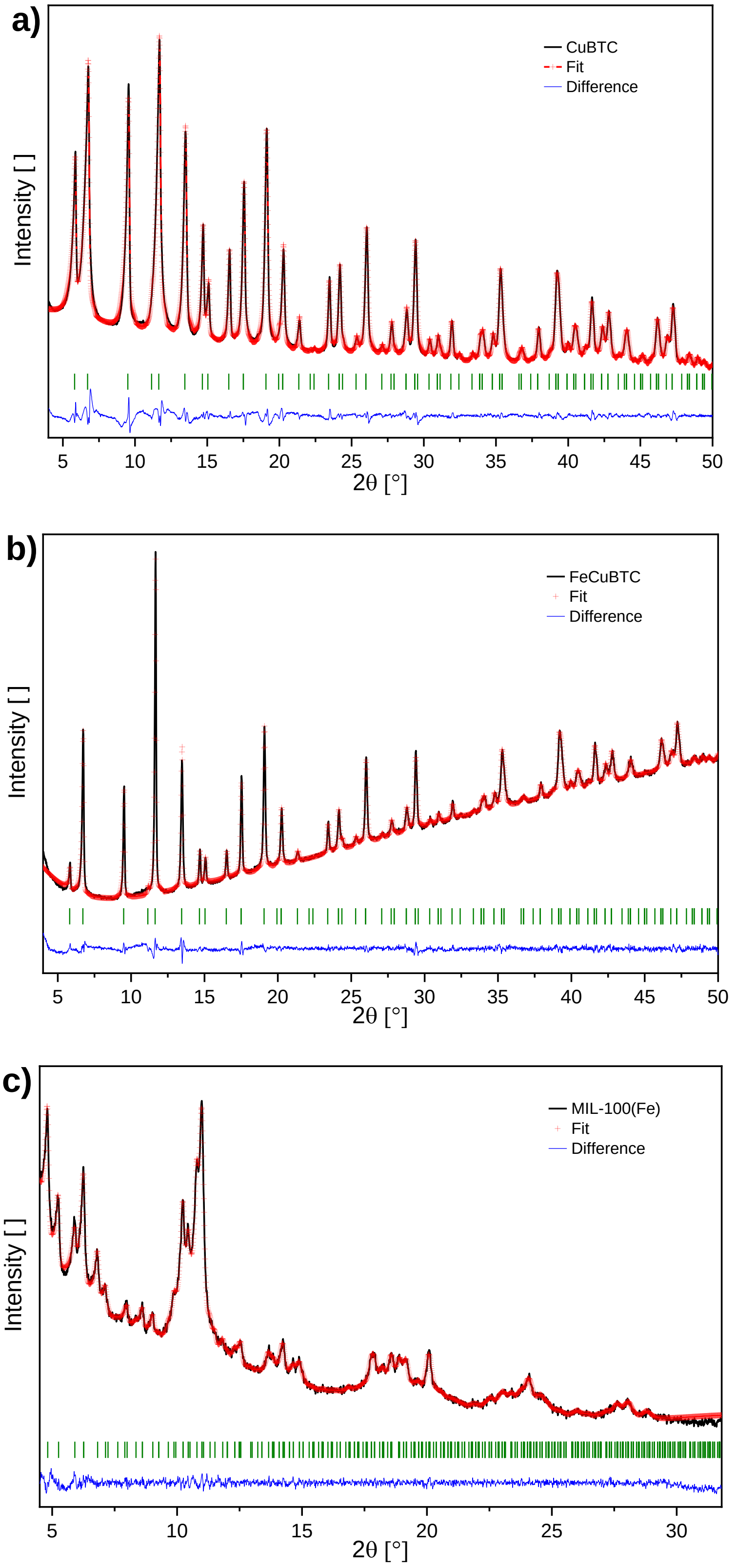

Figure S2. Results of structure refinements using Pawley fits for a) CuBTC, b) FeCuBTC and c) MIL-100(Fe).

Based on single crystal structure data for monometallic FeBTCs with HKUST-1 structure, a more pronounced expansion of the unit cell was initially expected for the incorporation of iron. The reason for this effect might be ascribed to the metal-metal interaction of copper and iron, which is also obvious in the obtained Fe-Cu distance of bimetallic paddlewheel units obtained from EXAFS analysis, but further studies are needed to gain detailed insights.

Table S1. Comparison of the unit cell parameters obtained from performed Pawley fits with literature values based on single crystal X-ray diffraction.

sample	Unit cell parameter a		Space group	R_{wp}
	Obtained from Pawley fit	Obtained from single crystal data		
FeCuBTC	26.323	-	$Fm\bar{3}m$	1.94
CuBTC	26.313	26.343[3]	$Fm\bar{3}m$	3.43
Fe(II-III)BTC	-	26.633[4]	$Fm\bar{3}m$	-
Fe(III)BTC	-	26.669[5]	$Fm\bar{3}m$	-

4. Metal ratios

Table S2. Initial and resulting Fe:Cu ratios of bimetallic Fe-Cu-BTC samples determined by ICP-OES.

sample	Fe : Cu ratio	
	Used during synthesis	Determined by ICP-OES
CuBTC/Fe_2O_3	30 : 70	39 : 61
CuBTC/F300	30 : 70	41 : 59
CuBTC/MIL-100(Fe)	30 : 70	32 : 68
FeCuBTC	30 : 70	31 : 69

5. Nitrogen physisorption

The type I physisorption isotherms of the bimetallic samples, which are characteristic for microporous materials, showed a reduced nitrogen uptake compared to monometallic CuBTC (see **Figure S3**). The determined specific surface areas (BET method, see **Table S3**) corroborated this observation. Furthermore, a trend was visible within the bimetallic samples. The specific surface area of FeCuBTC was significantly lower than CuBTC, which might be attributed to defects created by the incorporation of iron. The even lower specific surface area of CuBTC/Fe_2O_3 compared to FeCuBTC was obvious, since the presence of α-Fe_2O_3 with a low surface area reduced the total surface area. On the other hand, the larger specific surface area of F300 and MIL-100(Fe) in combination with a bimetallic FeCuBTC structure led to slightly larger total surface areas for CuBTC/F300 and CuBTC/MIL-100(Fe) compared to FeCuBTC.

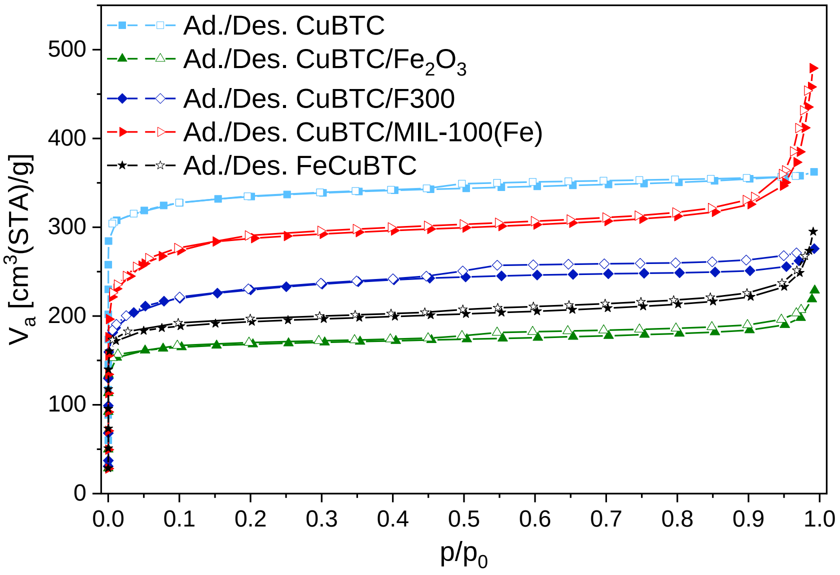

Figure S3. Nitrogen physisorption isotherms showed a changed adsorption behavior compared to CuBTC upon incorporation of iron in the bimetallic samples.

Table S3. Specific surface areas obtained by the BET method.

Sample	CuBTC	CuBTC/Fe_2O_3	CuBTC/F300	CuBTC/MIL-100(Fe)	FeCuBTC
S_{BET} [m²/g]	1310	670	870	1070	760

6. Reaction monitoring of the synthesis of a CuBTC/F300 material

A closer analysis of the reaction course provided a plausible explanation for the higher iron content in CuBTC/F300. At the beginning of the reaction, a dark brown solid formed, which turned greenish after several hours. A time-dependent study on a similar sample showed that an F300 phase was formed initially, and after 20 minutes, first reflections of an HKUST-1 structure were visible (see **Figure S4**). The F300 phase consisted mostly of iron and only the formation of the HKUST-1 framework led to a higher copper content. Overall, for CuBTC/F300 all of the iron seemed to be consumed during the formation of a F300 phase in the beginning and due to the excess of metals compared to linker, not all of the copper could be incorporated into the material resulting in the observed Fe:Cu ratio of 41:59.

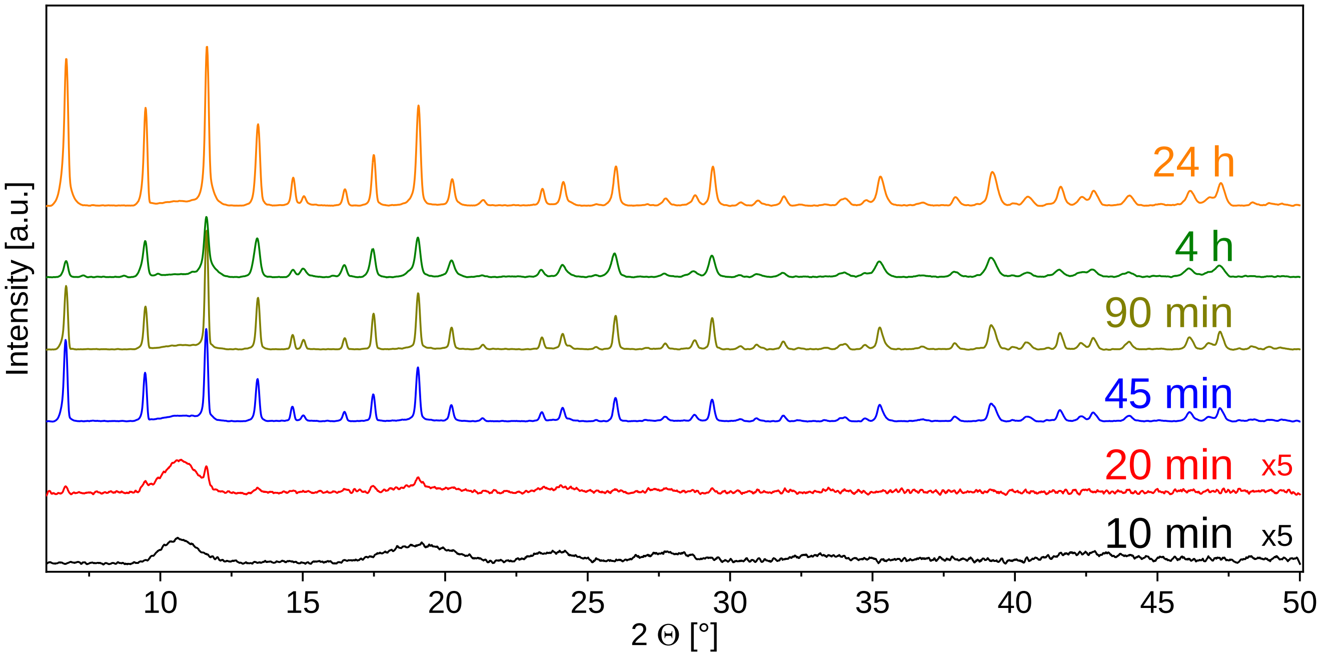

Figure S4. Powder X-ray diffraction pattern of samples, which were synthesized in the same fashion as CuBTC/F300, but with a feeding ratio of Fe:Cu = 20:80. The syntheses were identical, but stopped after various reaction times between 10 min to 24 h. At the beginning, only broad reflections of an F300 phase were visible. Starting from 20 min, first reflections of the HKUST-1 structure were observed.

Table S4. Determined Fe : Cu ratios for a CuBTC/F300 sample with a feeding ratio of Fe:Cu = 20:80 stopped after various reaction times. The ratios were determined by using a Z-6100 Polarized Zeeman atomic absorption spectrometer from Hitachi. The samples were digested in 2 mol/L HCl solution for the analysis.

**Reaction time**	**10 min**	**20 min**	**45 min**	**90 min**	**4 h**	**24 h**
**Fe : Cu ratio**	94 : 6	81 : 19	47 : 53	38 : 62	28 : 72	28 : 72

7. DFT-based frequency analysis of IR bands

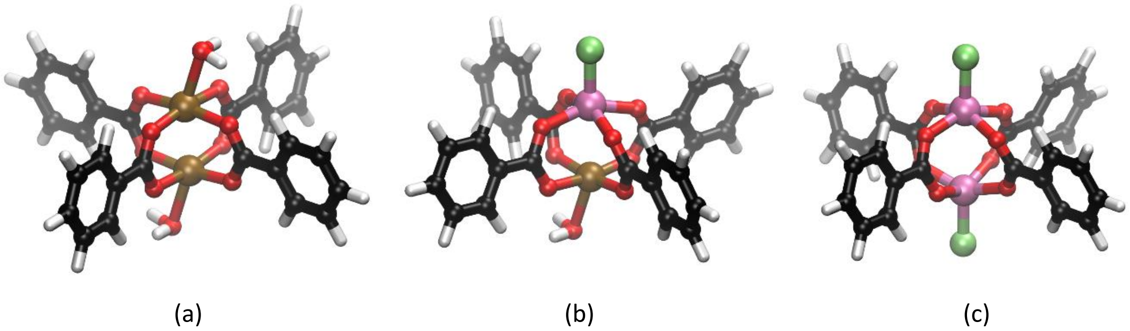

Figure S5. DFT optimized structures of the three paddle-wheel models.

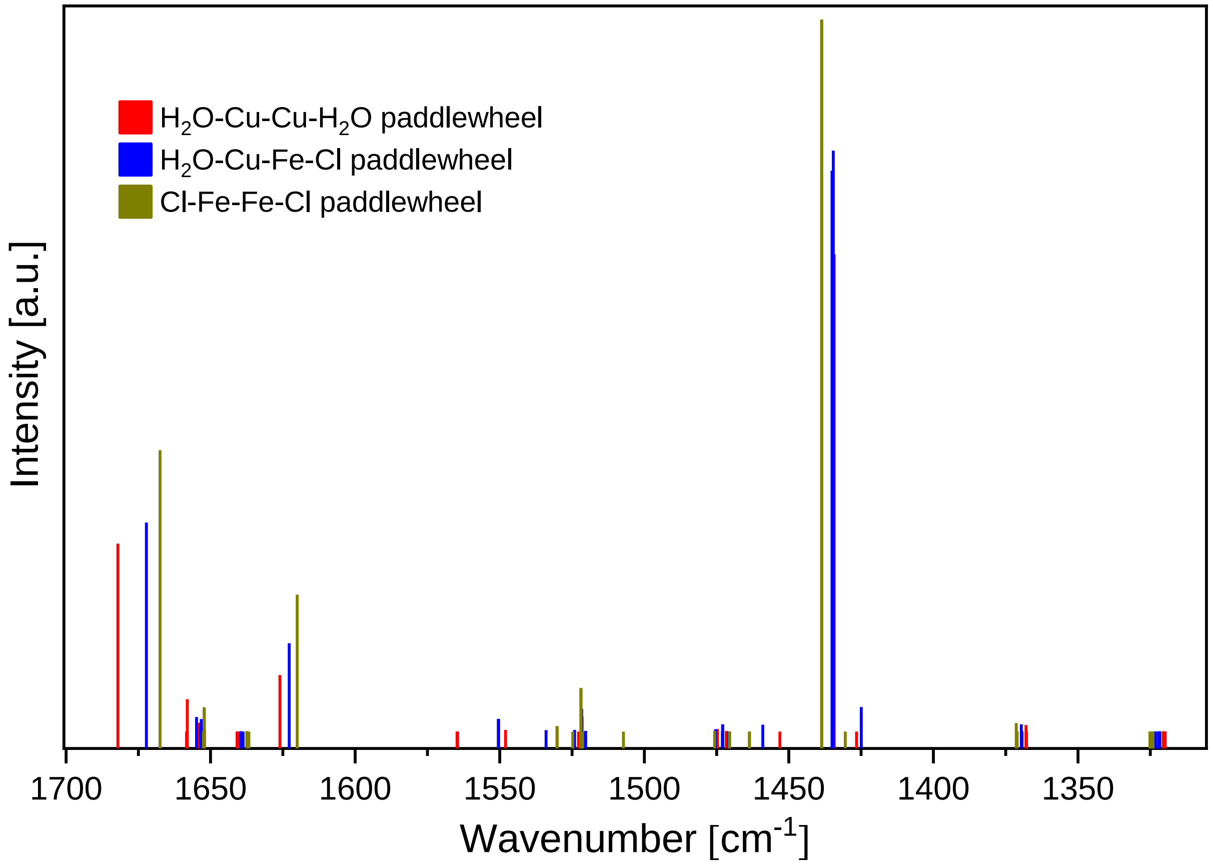

Figure S6. Calculated band positions and relative intensities obtained from the DFT-based frequency analysis.

8. EXAFS fit results

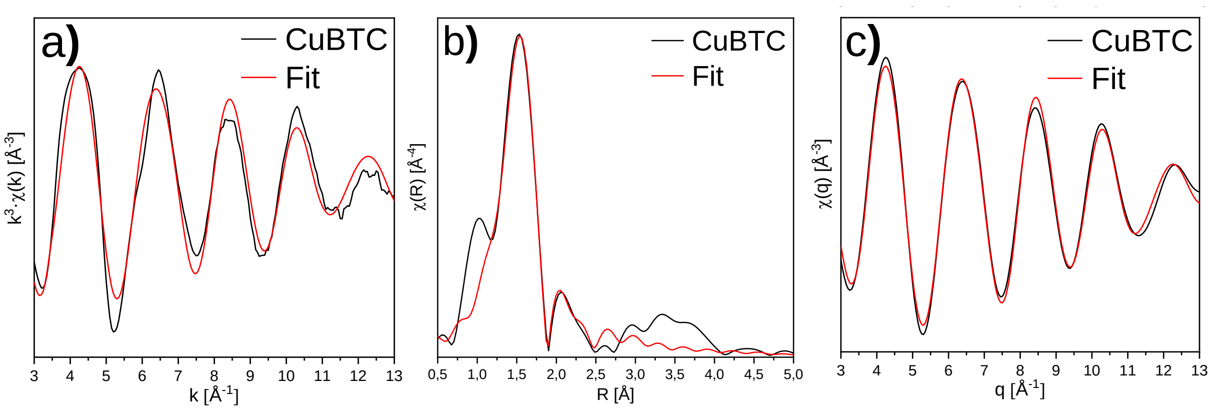

Figure S7. EXAFS spectrum (a), Fourier-transformed EXAFS spectrum (b) and the back Fourier-transformed spectrum (c) of CuBTC recorded at the Cu K-edge.

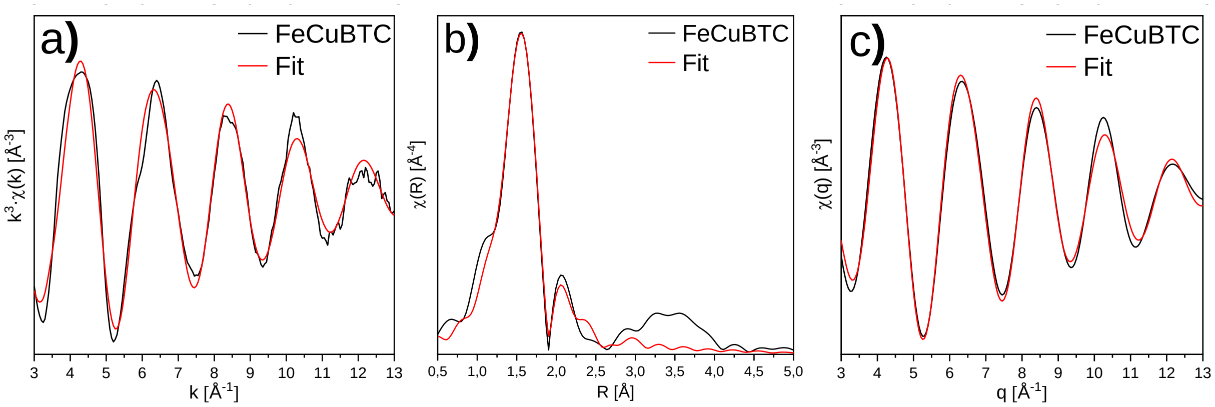

Figure S8. Experimental obtained Fourier-transformed EXAFS spectra and the best fit calculations of FeCuBTC at the Cu K-edge without iron neighbors.

Table S5. Best fit values for FeCuBTC at the Cu K-edge without iron neighbors.

Sample	Abs-Bs[a]	N(Bs)[b]	R(Abs-Bs)[c] [Å]	$\sigma^{2[d]}$ [Å²]
**FeCuBTC**	Cu – O	5	1.96 ± 0.02	0.006 ± 0.0004
Cu K-edge	Cu – Cu	1	2.65 ± 0.07	0.018 ± 0.0067
	Cu – C	4	2.85 ± 0.07	0.018 ± 0.0067
	Cu – O	4	3.09 ± 0.05	0.018 ± 0.0067
	$S_0^{2[e]}$ = 0.8892; $\Delta E_0^{[f]}$ = 6.73 ± 1.33 eV; χ^2_{red}[g] = 301; R[h] = 0.014; N(path)[i] = 4; N(par)[j] = 7; k-range: 3 - 13; R-range: 1.0 - 3.0.			

[a] Abs = X-ray absorbing atom, Bs = backscattering atom. [b] Number of backscattering atoms. [c] Distance between absorbing and backscattering atom. [d] Debye-Waller factor. [e] Amplitude reducing factor. [f] Accounts for the shift of E_0 between theory and experiment. [g] Reduced χ^2 error (considers the number of independent points and number of varied parameters besides the error to the experiment). [h] Fit index. [i] Total number of fitted paths including single and multiple scattering paths. [j] Number of free parameters used for the fit.

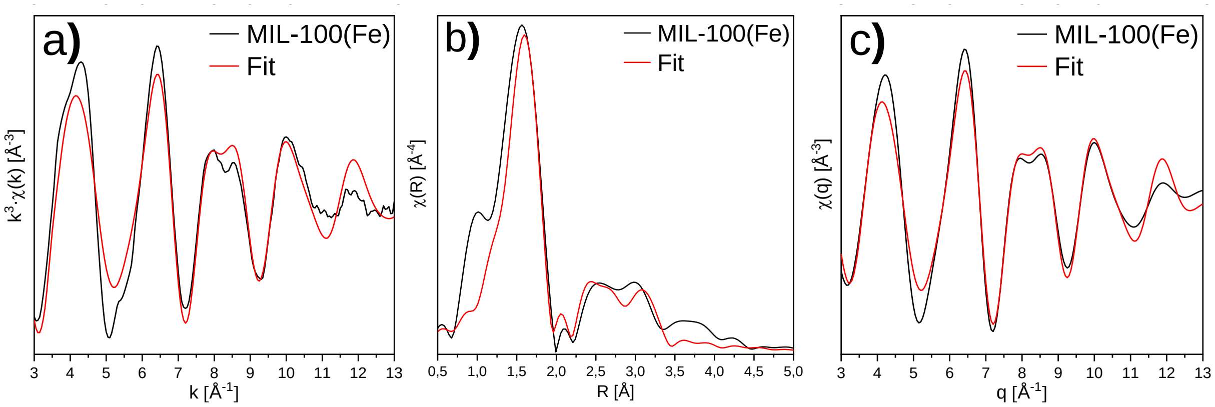

Figure S9. Experimental obtained Fourier-transformed EXAFS spectra and the best fit calculations of MIL-100(Fe) at the Fe K-edge.

Table S6. Best fit values for MIL-100(Fe) at the Fe K-edge was in accordance with reported single crystal data[6].

Sample	Abs-Bs[a]	N(Bs)[b]	R(Abs-Bs)[c] [Å]	$\sigma^{2[d]}$ [Å²]
**MIL-100(Fe)**	Fe – O	6	2.01 ± 0.02	0.0058 ± 0.0009
Fe K-edge	Fe – C	8	2.97 ± 0.04	0.0103 ± 0.0049
	Fe – Fe	2	3.37 ± 0.03	0.0084 ± 0.0035
	$S_0^{2[e]}$ = 0.7592; $\Delta E_0^{[f]}$ = 7.74 ± 2.39 eV; χ^2_{red}[g] = 2829; R[h] = 0.054; N(path)[i] = 3; N(par)[j] = 7; k-range: 3 - 13; R-range: 1.0 - 3.3.			

[a] Abs = X-ray absorbing atom, Bs = backscattering atom. [b] Number of backscattering atoms. [c] Distance between absorbing and backscattering atom. [d] Debye-Waller factor. [e] Amplitude reducing factor. [f] Accounts for the shift of E_0 between theory and experiment. [g] Reduced χ^2 error (considers the number of independent points and number of varied parameters besides the error to the experiment). [h] Fit index. [i] Total number of fitted paths including single and multiple scattering paths. [j] Number of free parameters used for the fit.

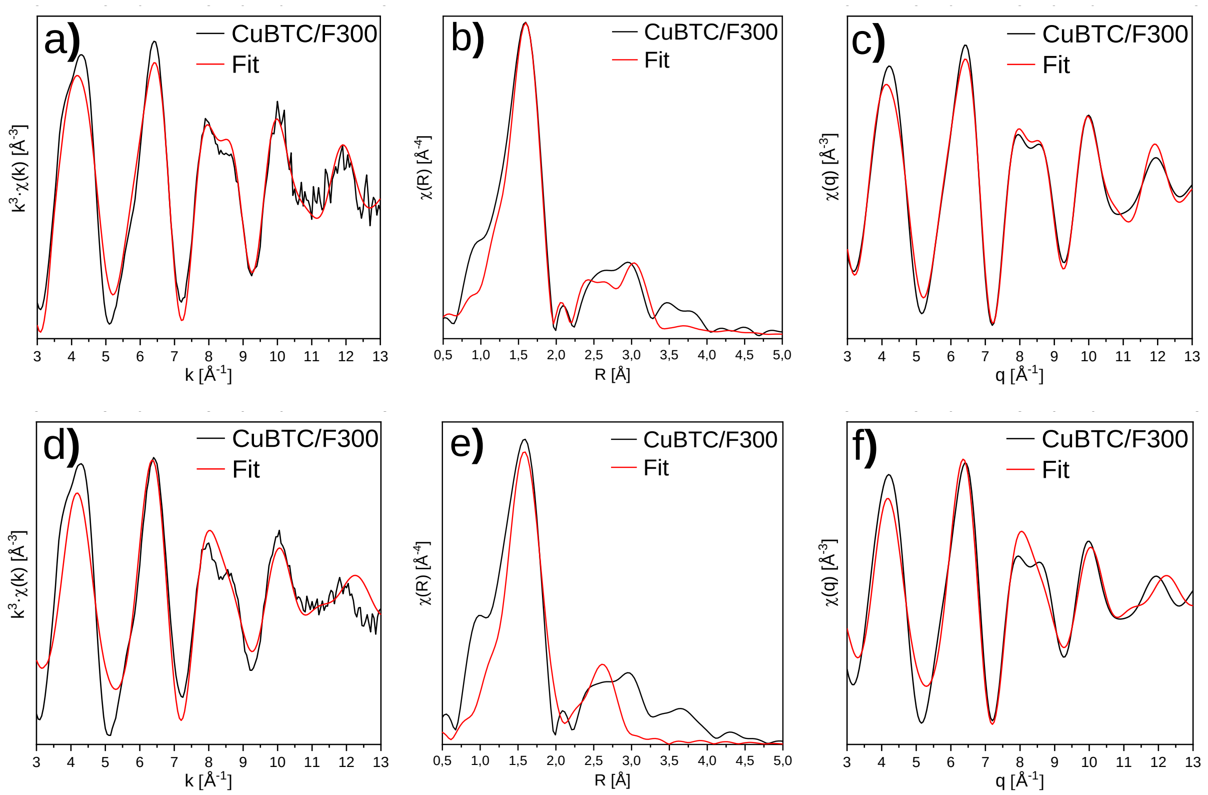

Figure S10. Experimental obtained Fourier-transformed EXAFS spectra and the best fit calculations of CuBTC/F300 at the Fe K-edge based on structural models of MIL-100(Fe) (**a-c**) and FeBTC (**d-f**).

Table S7. Best fit values for CuBTC/F300 at the Fe K-edge with either MIL-100(Fe) or FeBTC structure models.

Sample	Abs-Bs[a]	N(Bs)[b]	R(Abs-Bs)[c] [Å]	$\sigma^{2[d]}$ [Å²]
**CuBTC/F300**	Fe – O	6	2.01 ± 0.02	0.0064 ± 0.0005
Fe K-edge	Fe – C	8	2.99 ± 0.03	0.0122 ± 0.0035
MIL-100(Fe) structure model	Fe – Fe	2	3.34 ± 0.03	0.0086 ± 0.0019
	$S_0^{2[e]}$ = 0.7592; $\Delta E_0^{[f]}$ = 7.13 ± 1.40 eV; χ^2_{red}[g] = 57; R[h] = 0.019; N(path)[i] = 3; N(par)[j] = 7; k-range: 3 - 13; R-range: 1.0 - 3.3.			
**CuBTC/F300**	Fe – O	4	1.98 ± 0.02	0.004 ± 0.0014
Fe K-edge	Fe – Cl	1	2.24 ± 0.04	0.004 ± 0.0014
FeBTC structure model	Fe – C	4	2.95 ± 0.04	0.011 ± 0.0042
	Fe – Fe	1	2.96 ± 0.04	0.011 ± 0.0042
	Fe – O	4	3.25 ± 0.09	0.011 ± 0.0042
	$S_0^{2[e]}$ = 0.7592; $\Delta E_0^{[f]}$ = 0.96 ± 4.20 eV; χ^2_{red}[g] = 606; R[h] = 0.087; N(path)[i] = 5; N(par)[j] = 7; k-range: 3 - 13; R-range: 1.0 - 3.3.			

[a] Abs = X-ray absorbing atom, Bs = backscattering atom. [b] Number of backscattering atoms. [c] Distance between absorbing and backscattering atom. [d] Debye-Waller factor. [e] Amplitude reducing factor. [f] Accounts for the shift of E_0 between theory and experiment. [g] Reduced χ^2 error (considers the number of independent points and number of varied parameters besides the error to the experiment). [h] Fit index. [i] Total number of fitted paths including single and multiple scattering paths. [j] Number of free parameters used for the fit.

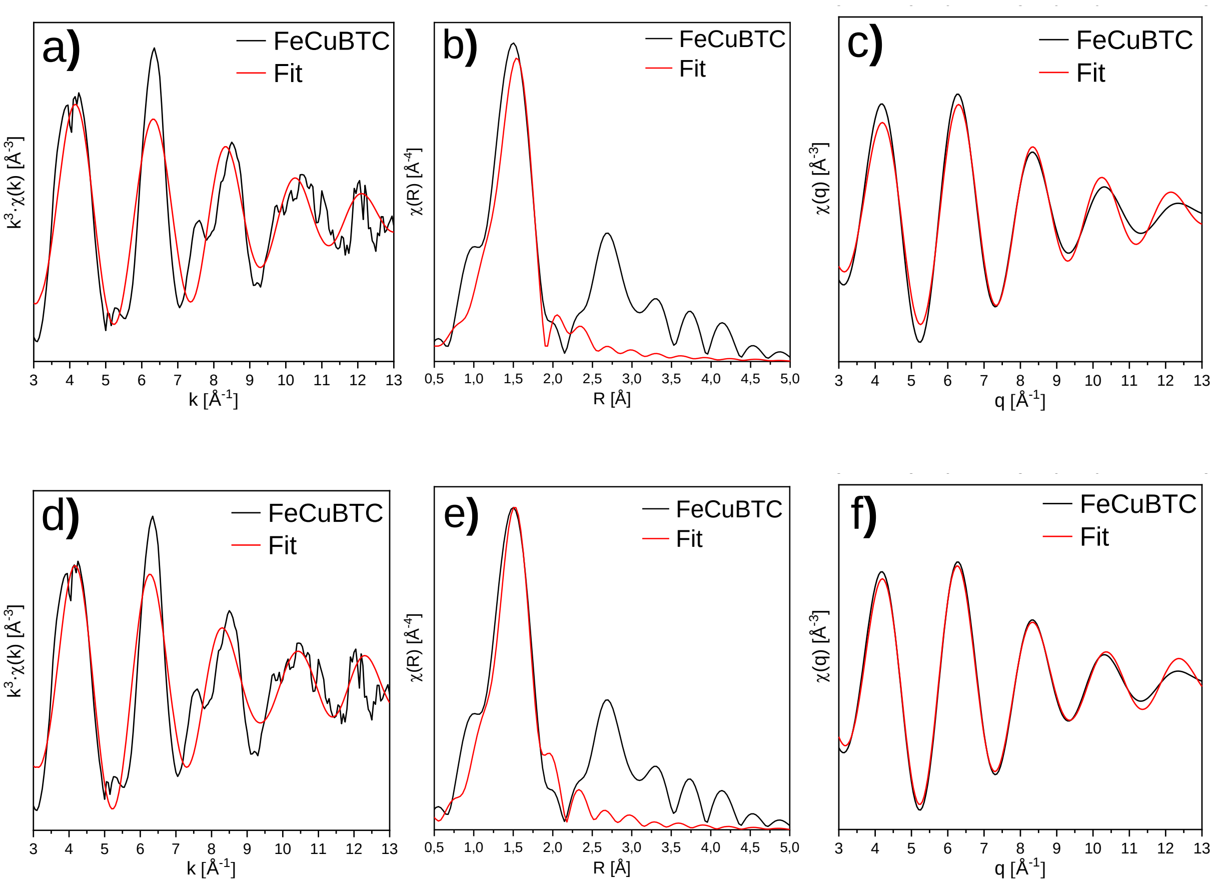

Figure S11. Experimental obtained Fourier-transformed EXAFS spectra and first shell fit calculations of FeCuBTC at the Fe K-edge with five oxygen only (**a-c**) and four oxygen and one chloride (**d-f**).

Table S8. First shell fit values for FeCuBTC at the Fe K-edge with either five oxygen or four oxygen and one chloride.

Sample	Abs-Bs[a]	N(Bs)[b]	R(Abs-Bs)[c] [Å]	$\sigma^{2[d]}$ [Å²]
**FeCuBTC**	Fe – O	5	4.98 ± 0.02	0.0074 ± 0.0010
Fe K-edge				
Five oxygen	S_0^2[e] = 0.7592; ΔE_0[f] = -0.43 ± 3.20 eV; χ^2_{red}[g] = 195; R[h] = 0.031; N(path)[i] = 1; N(par)[j] = 3; k-range: 3 - 13; R-range: 1.0 – 2.0.			
**FeCuBTC**	Fe – O	4	1.95 ± 0.02	0.0058 ± 0.0009
Fe K-edge	Fe – Cl	1	2.26 ± 0.02	0.0085 ± 0.0035
Four oxygen + one chloride	S_0^2[e] = 0.7592; ΔE_0[f] = -2.53 ± 2.78 eV; χ^2_{red}[g] = 152; R[h] = 0.017; N(path)[i] = 2; N(par)[j] = 4; k-range: 3 - 13; R-range: 1.0 – 2.0.			

[a] Abs = X-ray absorbing atom, Bs = backscattering atom. [b] Number of backscattering atoms. [c] Distance between absorbing and backscattering atom. [d] Debye-Waller factor. [e] Amplitude reducing factor. [f] Accounts for the shift of E_0 between theory and experiment. [g] Reduced χ^2 error (considers the number of independent points and number of varied parameters besides the error to the experiment). [h] Fit index. [i] Total number of fitted paths including single and multiple scattering paths. [j] Number of free parameters used for the fit.

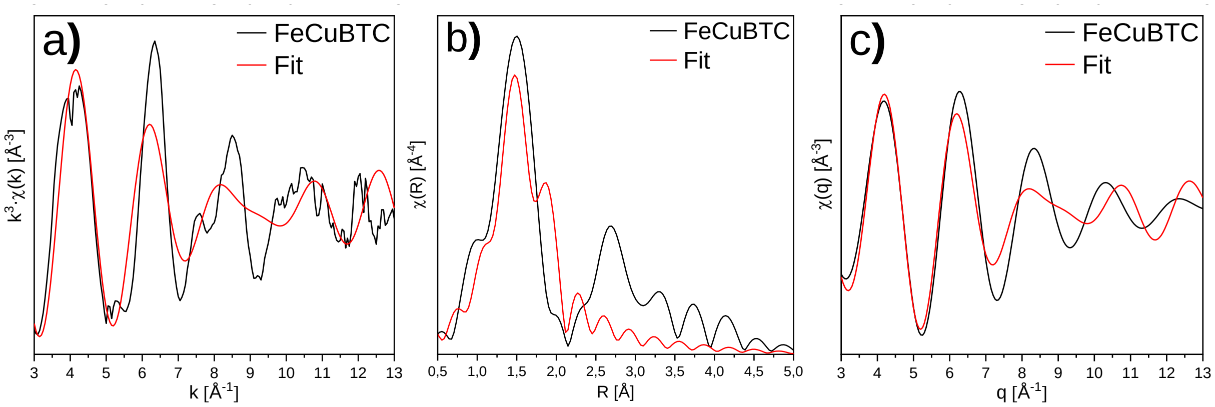

Figure S12. Experimental obtained Fourier-transformed EXAFS spectra and the best fit calculations of FeCuBTC at the Fe K-edge with the first two oxygen shells from α-Fe_2O_3.

Table S9. Best fit values for FeCuBTC at the Fe K-edge with the first two oxygen shells from α-Fe_2O_3.

Sample	Abs-Bs[a]	N(Bs)[b]	R(Abs-Bs)[c] [Å]	$\sigma^{2[d]}$ [Å²]
**FeCuBTC**	Fe – O	3	1.95 ± 0.02	0.0038 ± 0.0029
Fe K-edge	Fe – O	3	2.12 ± 0.02	0.0038 ± 0.0029
α-Fe_2O_3	$S_0^{2[e]}$ = 0.7592; $\Delta E_0^{[f]}$ = 3.20 ± 6.06 eV; $\chi^2_{red}{}^{[g]}$ = 887; R[h] = 0.14; N(path)[i] = 2; N(par)[j] = 3; k-range: 3 - 13; R-range: 1.0 – 2.0.			

[a] Abs = X-ray absorbing atom, Bs = backscattering atom. [b] Number of backscattering atoms. [c] Distance between absorbing and backscattering atom. [d] Debye-Waller factor. [e] Amplitude reducing factor. [f] Accounts for the shift of E_0 between theory and experiment. [g] Reduced χ^2 error (considers the number of independent points and number of varied parameters besides the error to the experiment). [h] Fit index. [i] Total number of fitted paths including single and multiple scattering paths. [j] Number of free parameters used for the fit.

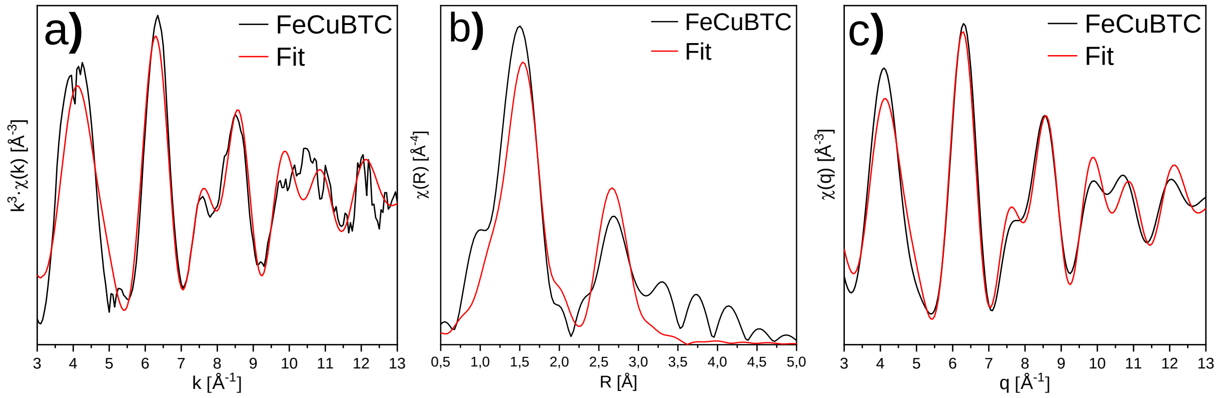

Figure S13. Experimental obtained Fourier-transformed EXAFS spectra and the best fit calculations of FeCuBTC at the Fe K-edge without copper neighbors.

Table S10. Best fit values for FeCuBTC at the Fe K-edge without copper neighbors.

Sample	Abs-Bs[a]	N(Bs)[b]	R(Abs-Bs)[c] [Å]	$\sigma^{2[d]}$ [Å²]
**FeCuBTC**	Fe – O	4	1.97 ± 0.02	0.0071 ± 0.0010
Fe K-edge	Fe – Cl	1	2.28 ± 0.05	0.0103 ± 0.0049
	Fe – C	4	3.00 ± 0.03	0.0071 ± 0.0010
	Fe – Fe	1	3.00 ± 0.03	0.0071 ± 0.0010
	Fe – O	4	3.31 ± 0.05	0.0071 ± 0.0010
	$S_0^{2[e]}$ = 0.7592; $\Delta E_0^{[f]}$ = -1.03 ± 3.97 eV; χ^2_{red}[g] = 208; R[h] = 0.055; N(path)[i] = 5; N(par)[j] = 7; k-range: 3 - 13; R-range: 1.0 - 3.1.			

[a] Abs = X-ray absorbing atom, Bs = backscattering atom. [b] Number of backscattering atoms. [c] Distance between absorbing and backscattering atom. [d] Debye-Waller factor. [e] Amplitude reducing factor. [f] Accounts for the shift of E_0 between theory and experiment. [g] Reduced χ^2 error (considers the number of independent points and number of varied parameters besides the error to the experiment). [h] Fit index. [i] Total number of fitted paths including single and multiple scattering paths. [j] Number of free parameters used for the fit.

9. EPR spectroscopy results

Table S11. Room temperature electron paramagnetic resonance (EPR) spectroscopy data.

**Sample**	**Intensity from double integrated EPR spectrum**	**Power [mW]**	**Intensity by square root of power**	**Mass [mg]**	**Intensity/ mass [A]**	**Relative total EPR signal intensities**	**Isotropic 'g' at RT**	**Linewidth ΔB_{pp} (mT) at RT**
CuBTC	2.8	2.0	2.0	0.0128	156	1	2.149	80.0
CuBTC/Fe_2O3	1.9	0.2	4.2	0.0090	467	3.1	2.130	35.0
CuBTC/MIL-100(Fe)	3.9	2.0	2.7	0.0100	270	1.7	2.102	83.0
FeCuBTC	9.7	0.2	21.7	0.0147	1476	9.5	2.023	36.0

10. References

[1] S. Marx, W. Kleist, A. Baiker, *J. Catal.* **2011**, *281*, 76.

[2] a) K. S. Walton, R. Q. Snurr, *J. Am. Chem. Soc.* **2007**, *129*, 8552; b) S. Brunauer, P. H. Emmett, E. Teller, *J. Am. Chem. Soc.* **1938**, *60*, 309.

[3] S. S.-Y. Chui, S. M.-F. Lo, J. P. H. Charmant, A. G. Orpen, I. D. Williams, *Science* **1999**, *283*, 1148.

[4] L. Xie, S. Liu, C. Gao, R. Cao, J. Cao, C. Sun, Z. Su, *Inorg. Chem.* **2007**, *46*, 7782.

[5] S. A. Sotnik, S. V. Kolotilov, M. A. Kiskin, Z. V. Dobrokhotova, K. S. Gavrilenko, V. M. Novotortsev, I. L. Eremenko, V. K. Imshennik, Y. V. Maksimov, V. V. Pavlishchuk, *Russ. Chem. Bull.* **2014**, *63*, 862.

[6] P. Horcajada, S. Surblé, C. Serre, D.-Y. Hong, Y.-K. Seo, J.-S. Chang, J.-M. Grenèche, I. Margiolaki, G. Férey, *Chem. Commun.* **2007**, 2820.
